# Supplementary material for: Acceptance and compliance with micronutrient powder (MNP) among children aged 6–23 months in northern Nigeria
Source: PLOS Glob Public Health. 2022 Oct 17;2(10):e0000961. doi: 10.1371/journal.pgph.0000961 (PMC10022258; doi:10.1371/journal.pgph.0000961)
Supplement: S1 Dataset — (ZIP) [file pgph.0000961.s008.zip › S8_Dataset/Spot Checks.zip!/Spot Checks/02072015_A_SO_AD_2_SC.pdf]

SPOT CHECK RECORDING FORM

State: Adamawa **LGA: ____Song____ Community: _Bolki_**

Name of caregiver (female): Agnes Jokton

Name or caregiver (male): ………………………

Sex of index child: _Female___ Age of index child (in months): _17 months_____________

Spot Check Information

1. **Date of MNP distribution to household** (circle option below based on LGA name above)**:**
   - **Kebbi:** BirninKebbi&Jega LGA: **June 16**
   - **Kebbi:** Danko-wasagu: **June 17**
   - **Adamawa:**Song, Ganye: **(June 18)**
   - **Adamawa:**Guyuk **June 19**
2. **Date of Spot Check Observation: ____2/7/2015_____**
3. **Number of sachets remaining**(Ask the caregiver to show you and count the specific number of sachets remaining)**:**

   44

4. **Number of sachets that should remainusing one MNP sachet per day** (Analyst only)**:**

   47

5. **Number of sachets remaining / number of sachets should be remaining**(Analyst only)**:**

_____44____ / ___47______ = _____93.6____ %

Probes

Question 1. You have **XX** MNP sachets remaining. Explain how easy or difficult it was to use the MNP product on a daily basis for your child 6 – 23 months? (Circle one answer)

Very Difficult **Difficult** **(Easy)** **Very Easy**

Question 2. Describe any specific challenges that you faced that made the MNP difficult to use on a daily basis?

There was no challenge. In the beginning days I had to forcefully feed the child the kunu that is mixed with MNP but gradually she is used to it now and she asks for the kunu herself.

Question 3. Describe specific aspects of the MNP product that you or your child enjoyed that made it easier to use on a daily basis?

Since I started giving her MNP I noticed that she has added weight and has better appetite. She eats more than before. I also noticed that she is healthier and has not had any illness since then. Breastfeeding has increased also and she has so much energy.

Question 4. What questions do you have about the MNP based on your experience using it?

What should I do if I miss a day of administering the MNP?

Question 5. What specific recommendations do you have that we can do as a program to make the MNP easier to use on a daily basis?

The only recommendation is to bring more of this product to our community.
